# Modeling the Quantitative Specificity of DNA-Binding Proteins from Example Binding Sites

**DOI:** 10.1371/journal.pone.0006736

**Published:** 2009-08-25

**Authors:** Dana S. F. Homsi, Vineet Gupta, Gary D. Stormo

**Affiliations:** Department of Genetics, Washington University School of Medicine, St. Louis, Missouri, United States of America; Institute of Infectious Disease and Molecular Medicine, South Africa

## Abstract

**Background:**

The binding of transcription factors to their respective DNA sites is a key component of every regulatory network. Predictions of transcription factor binding sites are usually based on models for transcription factor specificity. These models, in turn, are often based on examples of known binding sites.

**Methodology/Principal Findings:**

Collections of binding sites are obtained in simulation experiments where the true model for the transcription factor is known and various sampling procedures are employed. We compare the accuracies of three different and commonly used methods for predicting the specificity of the transcription factor based on example binding sites. Different methods for constructing the models can lead to significant differences in the accuracy of the predictions and we show that commonly used methods can be positively misleading, even at large sample sizes and using noise-free data. Methods that minimize the number of predicted binding sequences are often significantly more accurate than the other methods tested.

**Conclusions/Significance:**

Different methods for generating motifs from example binding sites can have significantly different numbers of false positive and false negative predictions. For many different sampling procedures models based on quadratic programming are the most accurate.

## Introduction

Identifying transcription factor binding sites is a key step in the modeling of regulatory networks. Great advances in our understanding would ensue if we could accurately predict the regulatory sites within a genomic sequence. But very few transcription factors (TFs) have been experimentally characterized well enough to know which of the vast number of potential binding sites have sufficient binding affinity to be used as regulatory sites *in vivo*. Much more commonly a small collection of binding sites is obtained and from them a model for the TF's binding specificity is determined. Sometimes quantitative affinity measurements will be made for a subset of binding sites and then predictions of affinities to all sites are extrapolated based on a model [1]–[4]. More recently quantitative measurements have been applied to many more sequences in high-throughput approaches [5]–[8]. But even in those cases it is usually not practical to measure the affinity to all possible binding sites, of which there are 4^L^ for an L-long binding site. Rather some model is employed, such as assuming that the binding energy contributions for each position are additive and therefore a simple position weight matrix (PWM) is sufficient to predict the binding affinity of the TF to any sequence [9]. When simple additive models do not provide accurate predictions more complex models can be used and a variety of approaches have been proposed [10]–[19].

In most cases the specificity of TFs are inferred from collections of binding sites. Binding site information for several hundred TFs, based on several types of experimental reports, are available in databases such as TRANSFAC and JASPAR [20], [21]. One can also apply motif discovery tools to sets of sequences that are expected to be bound by a common TF to infer its specificity [22]. Those sequences may be derived from a variety of experimental approaches, such as genes with coordinated expression patterns [23], chromatin-immunoprecipitation (ChIP) experiments [24], [25] or even just collections of genes expressed in specific tissues [26]–[28]. Regardless of how the collection of binding sites is obtained, and especially important for the use of motif discovery methods, a model for the TF's specificity must be used, and usually it is some form of PWM. But there are several methods for determining a PWM from a set of binding sites, and they can lead to very different predictions. The most commonly used method is a log-odds approach in which the frequencies of bases in the binding sites are assumed to be proportional to their contributions to binding affinity [9], [22]. Another commonly used method is the Match program which is often used with the TRANSFAC database [29]. More recently two groups have proposed a method based on quadratic programming that seeks to minimize the number of unobserved sites that are predicted to be binding sites [30], [31].

In this paper we compare the intrinsic ability of different approaches to determine an accurate model for TF specificity from example binding sites. We employ a simulation study so that the correct model is known and we can generate noise-free datasets. We compare the accuracies of the different approaches while varying the sample sizes and the method for obtaining the samples. In many cases the quadratic programming method performs as well as or better than the other methods tested, even when sites are drawn from a Boltzmann distribution which is the assumption used in the log-odds approach.

## Results

**Figure 1:**
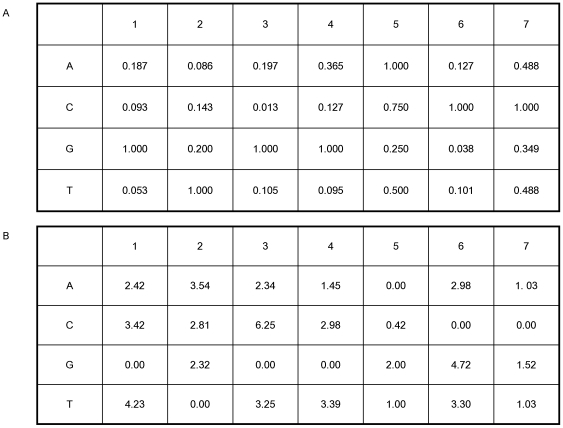
Mnt binding matrices. A. The experimentally observed relative frequency of each type of DNA base at each position in the 7-long Mnt protein half-site. The highest affinity site is: 5′-GTGGACC-3′. B. The negative log2 of the values shown in A. Each entry represents the binding energy contributed by a particular base at that position in the site to the total binding energy. This matrix represents the “true” binding model.

**Figure 2:**
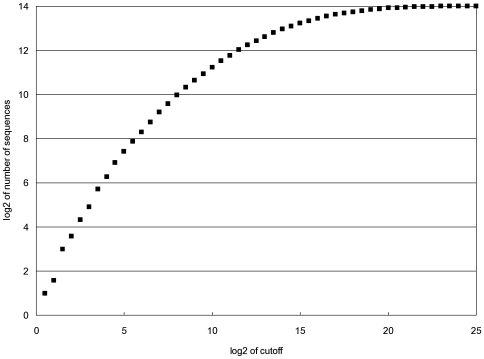
The log2 of the number of sequences in the population of all DNA 7-mers that are below or equal to the relative binding energy indicated on the x-axis. The “log2 of cutoff” is a DNA-protein binding energy based on the affinity values listed in Figure 1B.

We use the half-site for the Mnt protein to illustrate the ability of different methods to accurately determine binding models based on sets of example binding sites. Alternative models based on other DNA-binding proteins give very similar results (data not shown), so we focus on an in-depth analysis of just this one protein. Mnt is a repressor from phage P22 for which the binding specificity has been well characterized [3]. It binds as a tetramer to a symmetric binding site and, in earlier work, all single base changes to the consensus site were synthesized and their change in binding affinity measured. Figure 1A shows those relative affinity measurements for the 7-long half-site. As with many DNA-binding proteins, some positions are very specific, such as position 3 where the affinity of a C is reduced over 70-fold compared to the consensus G. Other positions have much smaller effects on affinity, such as position 7 where the largest effect is less than 3-fold between a G and the consensus C. Figure 1B contains the −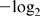 of those relative affinities, which are proportional to the difference in binding energy between the consensus base and each other base. Although the binding of Mnt to DNA is not strictly additive [32], in these simulations we assume that the binding energy to any sequence is the sum of the energy values from the matrix that corresponds to that sequence. This makes the true model conform to the assumption of additivity employed by each of the compared methods and avoids any confounding effects of correlated positions. If *E* is the energy matrix of Figure 1B, then the binding energy of any sequence *S_j_* is 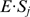 where *S_j_* is a matrix of the same form as *E* that contains a 1 for the base that occurs at each position and a 0 for all other bases at each position [9]. We further assume that there is some threshold of affinity that a site must have in order for it to function as a regulatory site *in vivo*. Figure 2 shows the number of sequences, from the total of 16,384 7-long DNA sequences, below various cutoffs in relative binding energy. As expected this follows an approximately exponential distribution for the high affinity (low energy) sites.

For cutoffs between 2 and 7 (affinities within 4-fold and 128-fold of the highest affinity site) we collected example sites in two different ways. In one we sampled from the sites below the cutoff assuming a Boltzmann distribution where the probability of a site being sampled is proportional to its binding affinity. In the other we sampled from a step function (an approximation to a Fermi-Dirac distribution with a steep transition) such that all sites below the threshold are equally likely to be sampled. The real distribution of sites *in vivo* will likely be between these two extreme cases. For each sampling method, and for each cutoff value from 2 to 7, we randomly selected sites with sample sizes of 20, 50 and 200. This covers the range of binding sites that are typically obtained experimentally and from which binding site models are generated. Each sampling procedure was performed five times and the mean and standard deviation of the means are reported.

**Figure 3:**
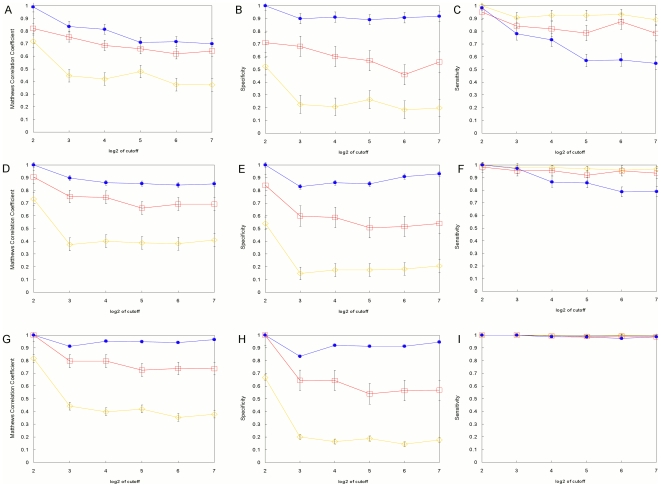
Performance of the position weight matrix models with step-function sampling. A–C. Alignments containing 20 sites. D–F. Alignments containing 50 sites. G–I. Alignments containing 200 sites. The log-odds, Match, and quadratic programming results are denoted by the red open-square, yellow open-diamond, and blue filled-oval markers respectively. Each data point is the mean of five replicates. Error bars denote the standard deviation of the mean.

**Figure 4:**
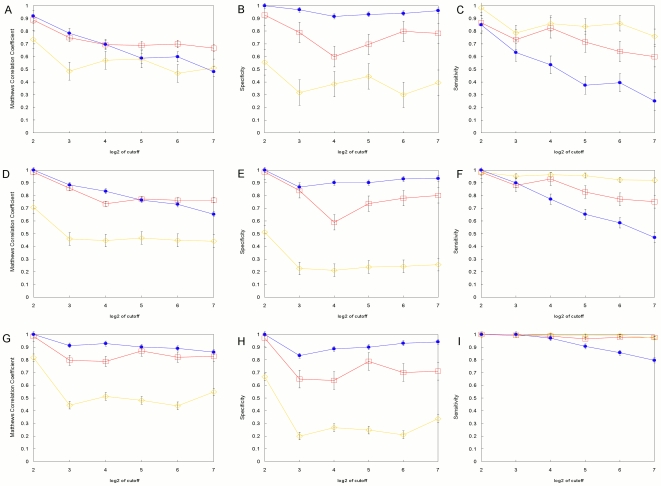
Performance of the position weight matrix models with Boltzmann sampling. A–C. Alignments containing 20 sites. D–F. Alignments containing 50 sites. G–I. Alignments containing 200 sites. The log-odds, Match, and quadratic programming results are denoted by the red open-square, yellow open-diamond, and blue filled-oval markers respectively. Each data point is the mean of five replicates. Error bars denote the standard deviation of the mean.

The three methods we use on the sets of example sites (see Methods) determine three different PWM scoring models, termed 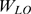 for the log-odds method, 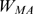 for the Match method, and 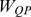 for the quadratic programming method. For these PWMs we use the bioinformatics convention that higher scores correspond to higher affinity, and therefore lower energy, sites (see Methods). Rather than trying to normalize and scale each model to a common standard, we compare them by simply choosing an equivalent cutoff score for each one and determining the number of false positives (FP) and false negatives (FN) compared to the true energy model. The cutoff for each model is set to the lowest scoring sequence in the example set from which the model is built. This assures that all of the observed binding sites are classified correctly and the FP (FN) sites are those that are misclassified because they score higher (lower) than the cutoff although their true binding affinity is lower (higher) than the cutoff. Figure 3 shows all of the results for the sites sampled using the step function. Parts A, B and C are the Matthews correlation coefficient (MCC, see Methods), the specificity and the sensitivity, respectively, for a sample size of 20 sites. Parts D-F and G-I are the same for sample sizes of 50 and 200, respectively. Figure 4 shows all of the results for sites sampled using the Boltzmann distribution, arranged the same as in Figure 3.

When sites are drawn from the step function distribution (Figure 3) 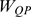 performs the best, by the MCC criterion, at every cutoff and for every sample size. This is not surprising because the 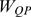 method essentially assumes that distribution, where all sites that are “good enough” are equally likely to be selected. It minimizes the number of sites not in the training set (the example sites from which the model is built) that are predicted to be sites, leading to the highest specificity. This results in somewhat lower sensitivity, although that improves at larger sample sizes.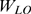 and 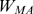 are worse by the criterion of MCC because they have lower specificity.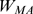 has the highest sensitivity because it makes the most predictions, many of which are false positives as shown by the lowest specificity and MCC. Neither 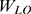 nor 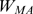 show much improvement in MCC at larger sample sizes because they assume the sites are drawn from a Boltzmann distribution, with higher affinity sites being more frequent in the observations. If binding sites *in vivo* are at or near saturating conditions, as has been suggested [30], then the Boltzmann assumption is incorrect and we can expect the 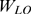 and 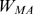 models based on example binding sites to be misleading even with large sample sizes, as we observe here.

When sites are drawn from a Boltzmann distribution, while still imposing a cutoff for the minimum affinity allowed (Figure 4), the results are more complex. For low cutoff values 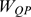 is still the best by MCC, but at higher cutoffs 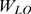 can be the best, although at large sample sizes they perform nearly the same. If we went to even higher cutoffs, 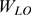 would be the best because the distributions would match more closely to the assumed complete Boltzmann distribution, but we expect that functional sites *in vivo* are constrained to be within some range of the optimum affinity, and a range of 128-fold lower seems reasonable. 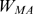 again has the highest sensitivity at every cutoff and sample size, but this is at the expense of many false positives so that it has the lowest specificity and MCC which are not improved with larger sample sizes.

**Figure 5:**
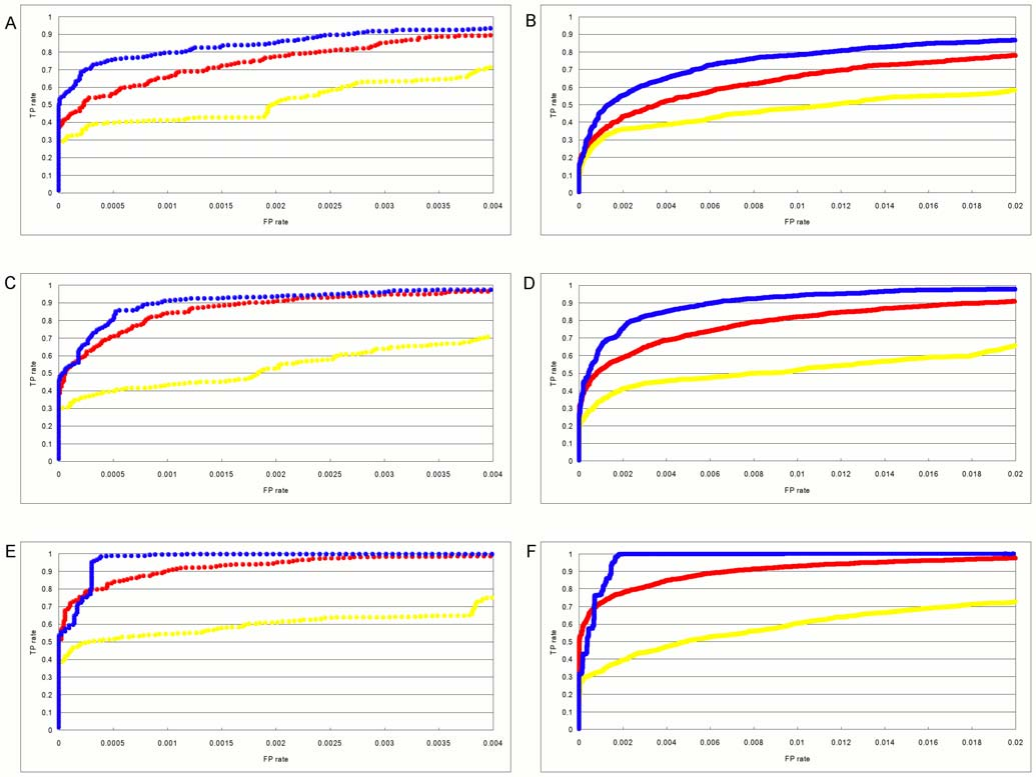
Receiver operator characteristic curves of the position weight matrix models with step-function sampling. The log-odds, Match, and quadratic programming results are denoted by red, yellow, and blue markers respectively. Each data point is the mean of five replicates and points are allowed to overlap. Error as standard deviation of the mean extends from each curve by the size of a single marker. A. Alignments containing 20 sites; cutoff = 4. The log-odds, Match, and quadratic programming curves attain TP = 1 at FP = 0.013, 0.099, and 0.019, respectively. B. Alignments containing 20 sites; cutoff = 7. The log-odds, Match, and quadratic programming curves attain TP = 1 at FP = 0.329, 0.495, and 0.200, respectively. C. Alignments containing 50 sites; cutoff = 4. The log-odds, Match, and quadratic programming curves attain TP = 1 at FP = 0.014, 0.057, and 0.011, respectively. D. Alignments containing 50 sites; cutoff = 7. The log-odds, Match, and quadratic programming curves attain TP = 1 at FP = 0.151, 0.437, and 0.070, respectively. E. Alignments containing 200 sites; cutoff = 4. The log-odds, Match, and quadratic programming curves attain TP = 1 at FP = 0.009, 0.038, and 0.006, respectively. F. Alignments containing 200 sites; cutoff = 7. The log-odds, Match, and quadratic programming curves attain TP = 1 at FP = 0.061, 0.253, and 0.013, respectively.

**Figure 6:**
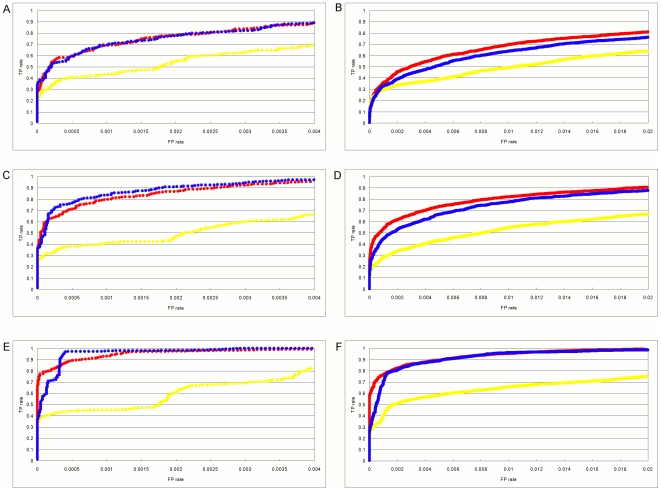
Receiver operator characteristic curves of the position weight matrix models with Boltzmann sampling. The log-odds, Match, and quadratic programming results are denoted by red, yellow, and blue markers respectively. Each data point is the mean of five replicates and points are allowed to overlap. Error as standard deviation of the mean extends from each curve by the size of a single marker. A. Alignments containing 20 sites; cutoff = 4. The log-odds, Match, and quadratic programming curves attain TP = 1 at FP = 0.026, 0.072, and 0.026, respectively. B. Alignments containing 20 sites; cutoff = 7. The log-odds, Match, and quadratic programming curves attain TP = 1 at FP = 0.238, 0.445, and 0.326, respectively. C. Alignments containing 50 sites; cutoff = 4. The log-odds, Match, and quadratic programming curves attain TP = 1 at FP = 0.011, 0.045, and 0.009, respectively. D. Alignments containing 50 sites; cutoff = 7. The log-odds, Match, and quadratic programming curves attain TP = 1 at FP = 0.107, 0.306, and 0.143, respectively. E. Alignments containing 200 sites; cutoff = 4. The log-odds, Match, and quadratic programming curves attain TP = 1 at FP = 0.008, 0.018, and 0.003, respectively. F. Alignments containing 200 sites; cutoff = 7. The log-odds, Match, and quadratic programming curves attain TP = 1 at FP = 0.035, 0.209, and 0.065, respectively.

The exact number of FPs and FNs depends on what scoring threshold is used to predict sites as positives and negatives. To further assess the accuracy of the PWM models when the prediction threshold is varied, we have plotted them as receiver operator characteristic (ROC) curves (Figures 5,6). In a ROC diagram, the TP rate, or sensitivity, is plotted on the y-axis against FP rate (or false discovery rate) on the x-axis as the prediction threshold is varied over all possible values. The ROC curve for a perfect predictor will go straight up to the point (0,1) and then over to (1,1), and any form of random guessing will place the curve along the diagonal of the ROC plot. This plot lets one compare methods for fixed values of FP or TP.

Figure 5 shows the ROC curves for models based on sites drawn from the step function. The left column is for a cutoff of 4 and the right column is for a cutoff of 7. Sample sizes of 20, 50 and 200 are plotted in the top, middle and bottom rows, respectively. As can be seen, 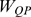 has the highest sensitivity at nearly every setting of FP rate for all sample sizes and both cutoffs. 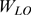 is usually somewhat worse, and 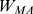 is always much worse. Figure 6 shows the same results for sites selected from the Boltzmann distribution. The 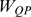 and 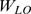 models are nearly the same for the lower cutoff of 4 (left column). At the higher cutoff of 7 (right column) the 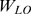 model is slightly better except at the largest sample size (bottom row) when they are again nearly the same. In all cases the 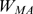 method performs considerably worse.

## Discussion

Since whole genome sequences have become available, one primary goal has been to identify the regulatory regions that are responsible for the control of gene expression. This often includes computational approaches to predict the binding sites of transcription factors, based on models for their specificity [22], [33]–[36]. Such methods, by themselves, suffer from high false positive rates so additional evidence, such as phylogenetic conservation, is sometimes used to improve the accuracy [37]. There can be many contributing factors to the high false positive rates, including the fact that many predicted sites may not be accessible *in vivo* and that TFs often function coordinately so that only sites in the correct context will be functional. But one significant contribution to false positives may arise from using a model, such as a PWM, that does not represent well the specificity of the TF. Since models for TF specificity are primarily generated from example binding sites, using optimal methods to estimate the specificity can be crucial in maximizing the accuracy of the predictions. In this paper we show that methods which minimize the total number of predicted sites, such as by the quadratic programming approach, can be much more accurate than other popular methods. If the example sites are drawn from a Boltzmann distribution, where sites are sampled in proportion to their affinity, then log-odds methods have similar accuracies. But it seems likely that the high affinity sites will be saturated, at least under some conditions [30], in which case the quadratic programming method provides the highest accuracy predictions. One characteristic that was not tested in this study is the sensitivity of the different methods to noisy data. The quadratic programming approach, because it finds the center of the example sites, can be very sensitive to erroneous data and even a single outlier in a large sample can have a large effect on the resulting model. Therefore it may be worthwhile to filter example sites to remove extremes that may be erroneous, but with that comes the risk of underestimating the true variability of the model. Because their models are based on the average of the example sites, in different ways, the log-odds and Match methods will be much less sensitive to erroneous data, especially at large sample sizes.

## Materials and Methods

### Binding site model

As a model for the specificity of a transcription factor we use the quantitative binding data obtained for the Mnt protein of phage P22 [3]. Mnt binds as a tetramer to a palindromic binding site. To keep the sites small for our simulations we only use the 7-long half-site. Figure 1A shows the binding affinities for all possible base substitutions at each position relative to the consensus site GTGGACC. Figure 1B shows the negative logarithms (to base 2) of the relative affinities, which are proportional to the binding energy differences. Although the actual Mnt protein has a modest amount of non-additivity [32], [38], for these simulations we assume that binding is completely additive. Therefore the energy matrix of Figure 1B allows us to calculate the change in binding energy, relative to the consensus sequence, for all possible 7-long sequences. Of course in real TFs we expect that there will be a plateau for non-specific binding at some low affinity value [39], [40], but since we are only interested in the high affinity sites, which could serve as regulatory sites *in vivo*, we ignore that and consider the energies determined by the matrix to be the true binding energies for all potential binding sites. Figure 2 shows the number of 7-long sites that are below a cutoff of binding energy for the full range of energies.

### Sets of example binding sites

Two different types of sets of example binding sites were generated for each cutoff score from 2 to 7. They correspond to sequences drawn from a step-function distribution or a Boltzmann distribution, in each case using a maximum energy cutoff for selected sites. In the step-function distribution all sites that fall below the cutoff are equally likely to be sampled. In the Boltzmann sampling the probability of a site being sampled is proportional to its relative affinity (Figure 1A). Therefore the sites with the highest affinity will be sampled most often, and those just below the cutoff will be sampled the least.

### Methods of model determination

We used three different methods to determine an energy matrix from the set of example binding sites. The most commonly used methods define the position weight matrix (PWM) as the log-odds of the observed frequencies of each base at each position compared to that expected by chance [9]. In these simulations we assume that the background is equiprobable, 0.25 for each of A, C, G and T. The PWM by this method takes the aligned binding sites and is calculated as:
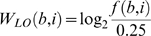
where *f(b,i)* is the frequency of each base, *b*, at each position, *i*, in the aligned binding sites. We add a pseudocount of 1 to observed counts to avoid frequencies of 0.

The second method we tested is from the Match program which is commonly used with the TRANSFAC database [29]. In this case the PWM is calculated as:
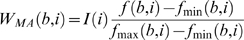



Where *I(i)* is the “information content” [41] at position *i*, defined as:
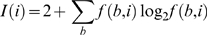
and *f_min_(b,i)* and *f_max_(b,i)* refer the minimum and maximum frequencies, respectively, that occur at position *i*. This method adds the frequencies of the bases at each position, scaled between 0 and 1 for the least to most frequent, and weighted by the information content of the position.

The third method is based on a quadratic programming approach presented recently by two different groups [30], [31]. In this method the PWM is found that scores all of the observed sequences above some constant (set to 1 here) while minimizing the total length of the PWM vector:
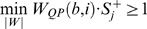
where 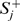 is the set of example binding sites (the positive dataset). Since the dot-product is related to the angle between the vectors, 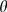, by:
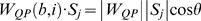
and since all of the sequence vectors are of the same length, minimizing |*W_QP_|* means maximizing 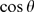, or minimizing 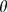, the angle between the PWM vector and all of the sequence vectors. Essentially this method is finding the PWM vector that is in the center of set of sequence vectors. This method is similar to training a support vector machine using only positive training data, to minimize the volume of sequence space that is allotted to positive scoring vectors [30], [31].

### Assessing the accuracy of the models

Each method for determining a PWM based on example sites has different score ranges and distributions, but their accuracies can be measured by determining the number of false positive and false negative predictions. 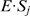 is the true binding energy for every sequence, and the predicted energy is 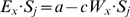, where *a* and *c* are offset and scaling factors and *x* refers to each different type of PWM. The important point is that ranking binding sites by their predicted energy from lowest to highest is equivalent to ranking them by their scores from highest to lowest. For a given cutoff of true binding energy we can determine every sequence that exceeds that cutoff and every sequence that falls below it. For each of the PWMs we set the cutoff to be the lowest scoring of the observed sequences, and then for all of the remaining sequences determine if they are above or below that cutoff. Sequences whose true value is above the cutoff but whose predicted value is below are false negatives, FN, and sequences whose true value is below the cutoff but whose predicted value is above are false positives, FP. The remaining sequences are correctly predicted true positives, TP, and true negatives, TN.

The Matthews correlation coefficient is a convenient method for combining all of the predictions into a single value:
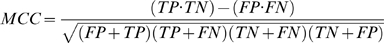



The value of MCC ranges from 1, where all predictions are correct, to −1, where all predictions are incorrect. Any type of random assignment leads to MCC = 0. Specificity is defined as TP/(TP+FP) and sensitivity is defined as TP/(TP+FN).

### Receiver operator characteristic curves

**Figure 7:**
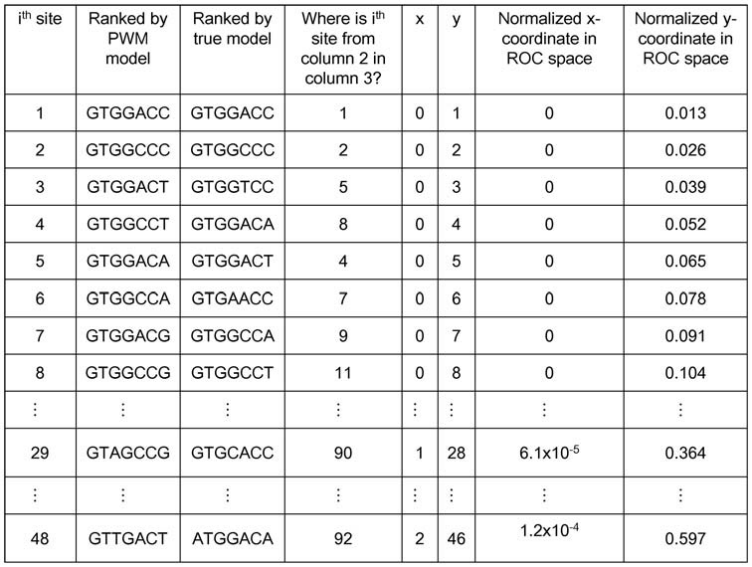
Creating a ROC curve. This figure shows a portion of a typical spreadsheet used to construct a ROC curve. In this example the PWM model is a log-odds model based on 50 sites. The cutoff is 4 and the sampling mode is Boltzmann. There are 77 sites whose true energy is below or equal to the cutoff (i.e. K = 77). In the original spreadsheet the index *i* runs from 1 to 16,384.

ROC curves were prepared with Microsoft Excel. The population of all 7mers was ranked by the true model from the lowest energy site to the highest. Similarly, a ranked list was prepared for each PWM model from highest to lowest scoring sites. Sites that had the same scores were allowed to be sorted by Excel and no modification of this ranking was attempted. The position of each PWM-ranked site in the true-ranked list was located and this information was saved as a “where is” list; see Figure 7. From Figure 2, the number of sites ranked at or above the threshold was known in each case and this value, K, was used in a comparison. Specifically, starting from the origin, points in ROC space were generated by moving down the “where is” list (column 4 in Figure 7) and executing this statement: if the “where is” number is greater than K, increment the x column by one, otherwise increment the y column by one. The x-axis was normalized by dividing through by 4^7^ –K; the y-axis was normalized by dividing through by K. The FP rate is represented by the x-axis, while the TP rate is represented by the y-axis.

### Availability of Software

All of the programs developed for this paper are available at ural.wustl.edu/QPLOMA. These include the programs for generating the samples, for generating each different type of model from the sampled sites, and for analysis based on MCC, specificity, sensitivity and ROC.
